# Artificial Intelligence of Things in Hydrogen Sensing: Toward Optic and Intelligent System

**DOI:** 10.34133/research.0750

**Published:** 2025-08-06

**Authors:** Jianxiong Zhu, Yifan Zhan, Xijie Ni, Yuze Gao

**Affiliations:** ^1^ School of Mechanical Engineering, Southeast University, Nanjing 211189, P. R. China.; ^2^ Guangdong Provincial Key Laboratory of Electronic Functional Materials and Devices, Huizhou, Guangdong 516007, P. R. China.; ^3^ Huizhou University, Huizhou 516001, P. R. China.; ^4^ Southeast University Suzhou Campus, Suzhou 215125, P. R. China.

## Abstract

Hydrogen sensing is of increasing importance in conjunction with the development and expanded utilization of hydrogen as an energy carrier or chemical reactant. This study focuses nanoscale hydrogen sensors that incorporate nanohybrid structural innovations to fabricate various systems, thereby enhancing detection efficiency and accuracy. Concurrently, advancements in optical hydrogen sensing and next-generation hybrid functional mechanisms have provided greater precision and universality with the aid of artificial intelligence of things (AIoT). For instance, optical hydrogen sensors offer high sensitivity and accurate gas detection with strong immunity to electromagnetic interference. Beyond optics, emerging models of next-generation composite multifunctional detection mechanisms provide operational advantages in hydrogen sensing, such as self-powering and long-range capabilities. In addition, the continual advancements in machine learning methods provide a feasible solution for data processing in hydrogen sensing applications through their integration with AIoT. This paper not only highlights the application of machine learning to enhance hydrogen sensor detection but also underscores its potential to improve the accuracy of future detection systems. In summary, these advances in nanohybrid structures, optical sensing, hybrid functional mechanisms, and machine learning integration represent strides in improving the performance, reliability, and versatility of hydrogen sensors, offering promising solutions for diverse hydrogen-related applications.

## Introduction

Hydrogen, as an efficient and clean energy source, has become crucial part of the future energy transition [1–3]. It shows great potential in reducing environmental pollution, decreasing greenhouse gas emissions, and replacing traditional fossil fuels. With the growing global demand for sustainable energy solutions, hydrogen technology has rapidly developed, and the application of hydrogen in fields such as energy [4], industry [5], and transportation [6] has gradually expanded. However, the safety management and monitoring of hydrogen are key to the widespread application of hydrogen energy [7,8]. The detection of hydrogen leakage is vital for ensuring human and environmental safety. Therefore, efficient and accurate hydrogen sensing technology has become an important research area [9,10]. The development of hydrogen sensors was initially motivated by the need for safe hydrogen use [11–13]. As hydrogen technology progresses and the expanding application scenarios of hydrogen, the demand for real-time monitoring of hydrogen has been growing. Hydrogen sensors can not only effectively monitor the concentration of hydrogen in the environment but also provide reliable technical means for hydrogen leak warning and accident prevention [14–17]. Based on different detection principles, hydrogen sensors can be categorized into several types, such as electrochemical [18], resistive, thermal conductivity, optical [19,20], and surface acoustic wave (SAW) sensors [21].

**Fig. 1.**
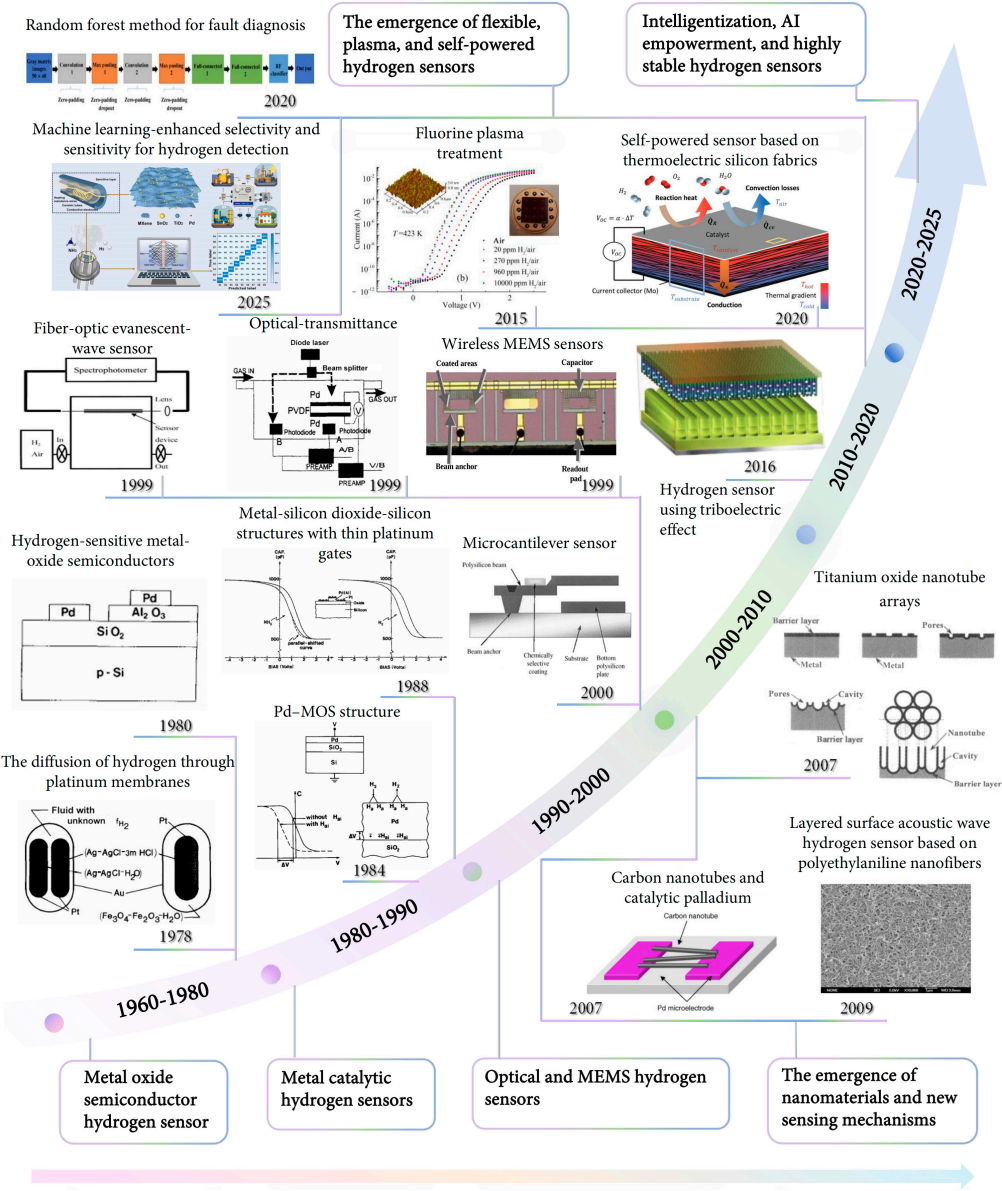
The milestone of era of AIoT in hydrogen sensing: from nanohybrid structure toward optic and intelligent system.

Hydrogen sensors have been widely used in multiple fields [17,22]. For example, in the energy sector, the safe operation of hydrogen fuel cells and hydrogen refueling stations relies on the support of hydrogen sensors. In industrial production, hydrogen sensors are used to monitor potential hydrogen leaks during the production process, ensuring production safety. In environmental monitoring, hydrogen sensors can measure the concentration of hydrogen in the atmosphere to assess environmental pollution. Furthermore, hydrogen sensors also play an important role in aerospace and military fields. With continuous advancements in science and technology, new materials such as graphene and metal–organic frameworks (MOFs) have been widely applied in the development of sensitive materials for hydrogen sensing, which improves the sensitivity, selectivity, and stability [23,24]. Moreover, combined with the latest wireless communication technology and the internet of things (IoT) technology [22], the developed wireless hydrogen sensing networks can achieve real-time monitoring of hydrogen concentration over large areas and multiple points, further improving the efficiency and accuracy of hydrogen leakage detection. Given the potential hazards associated with hydrogen leaks, key requirements have been established to ensure that these sensors operate effectively and safely. Typically, hydrogen sensors need to detect levels around 10 parts per million (ppm) to meet safety standards. Meanwhile, selectivity is another vital requirement to identify hydrogen without interference from other gases. Hydrogen-rich environments may contain gases like carbon monoxide, methane, and ammonia, which could interfere with gas identification. Moreover, the limit of detection (LoD) determines the lowest concentration of hydrogen that the sensor can accurately detect. Many applications require LoD capabilities as low as 0.1 ppm to comply with safety and environmental standards. Exposure to harsh environmental conditions or contaminants can degrade sensor performance, making stability a critical factor for consistent, reliable readings and for reducing the frequency of recalibrations or replacements. The milestone era of artificial intelligence of things (AIoT) in hydrogen sensing from nanohybrid structure toward optical and intelligent system can be found in Fig. 1. Hydrogen sensor development began in 1978 with research on hydrogen diffusion through platinum membranes at high temperatures and pressures, followed by the creation of a stable palladium-gate metal oxide semiconductor capacitor in 1981. In 1984, the Pd-MOS structure was applied to hydrogen sensing in catalytic reactions, and by 1988, metal–silicon dioxide–silicon structures were explored for hydrogen and ammonia detection. Entering the 21st century, wireless micro-cantilever chemical sensors based on micro-electrical and mechanical system (MEMS) technology were proposed in 1999–2000, alongside the development of a photoacoustic hydrogen sensor using laser amplitude modulation. Also in 2000, a hydrogen sensor based on optical fiber evanescent waves emerged, incorporating Pd-supported tungsten oxide as a sensitive material. Subsequent advances saw nanomaterials and novel structures gain prominence; for example, in 2007, a carbon nanotube–Pd catalyst interface was built via dielectrophoresis for hydrogen detection, and a surface acoustic wave hydrogen sensor using polyacetylene fiber was created, while titanium dioxide nanotube arrays were investigated for sensing. More recently, the focus shifted to self-powered and selective detection, with a triboelectric-based self-powered hydrogen sensor introduced in 2016 and a high-sensitivity thermoelectric silicon fabric sensor developed in 2021. Performance enhancements included fluorine plasma treatment for Pd/WO₃/SiC Schottky diode sensors in 2015, and artificial intelligence integration began, such as convolutional neural networks with random forests for sensor fault diagnosis in 2020, and a machine learning-enhanced SnO₂–TiO₂/MXene dual-gas sensor for hydrogen and ammonia detection proposed in 2025.

The growing demand for hydrogen across various sectors highlights the critical importance of sensor performance to overcome current limitations and meet evolving safety requirements. With the rise of hydrogen fuel cells in transportation, compact and energy-efficient sensors are increasingly required for onboard diagnostics. Additionally, higher sensitivity and selectivity are necessary to ensure safe operations in potentially explosive environments. Prolonging sensor lifespan while maintaining accuracy is crucial for industrial applications where maintenance costs are high. As modern sensors are increasingly integrated with IoT systems, real-time data integration is also necessary, allowing for instant data processing and remote monitoring capabilities. Ongoing research focuses on meeting these requirements by developing advanced materials like 2-dimensional (2D) nanostructures that offer higher sensitivity and selectivity. Artificial intelligence-enhanced algorithms are being explored to mitigate cross-sensitivity and predict sensor failures, which could greatly improve the reliability and efficiency of hydrogen sensors in a range of applications.

**Fig. 2.**
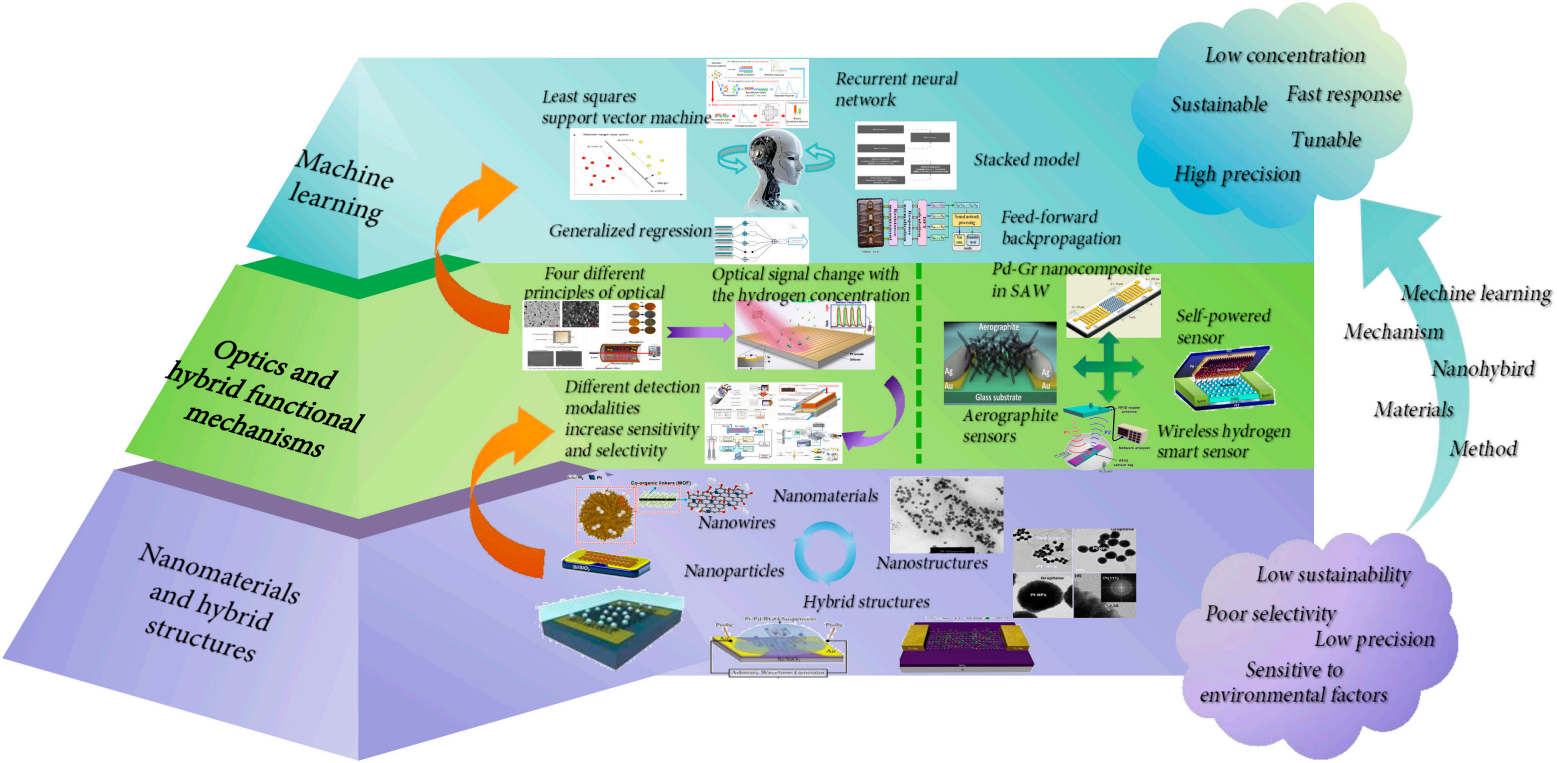
From nanohybrid structures to optics and more mechanism, and finally to era of AIoT system.

This paper conducts a comprehensive literature review from knowledge mapping analysis to identify current research hotspots, as shown in Fig. 2. It delineates progress across 4 pivotal areas: innovations in nanoscale architectures, optical effects applied to hydrogen sensors, next-generation composite functional detection mechanisms, and ML-enhanced methodologies. At the micro- and nanoscales, sensors incorporate hybrid structural innovations during fabrication, thereby augmenting the efficiency and accuracy. Meanwhile, the development of optical hydrogen sensors and next-generation functional mechanisms could further improve the accuracy and universality, due to their immunity to electromagnetic interference, high sensitivity, and excellent applicability in wide-range and sub-ppm detection. Simultaneously, next-generation hybrid mechanisms include emerging models for long-range wireless operation and industrial safety systems, breakthroughs in the design of low-power, ultra-light graphene sensors, self-powered systems, and zero-energy consumption monitoring. Furthermore, the development of modern ML has led to viable solutions for hydrogen sensor data processing and further improvement of accuracy with the aid of AIoT.

## Knowledge Graph of Hydrogen Sensor

**Fig. 3.**
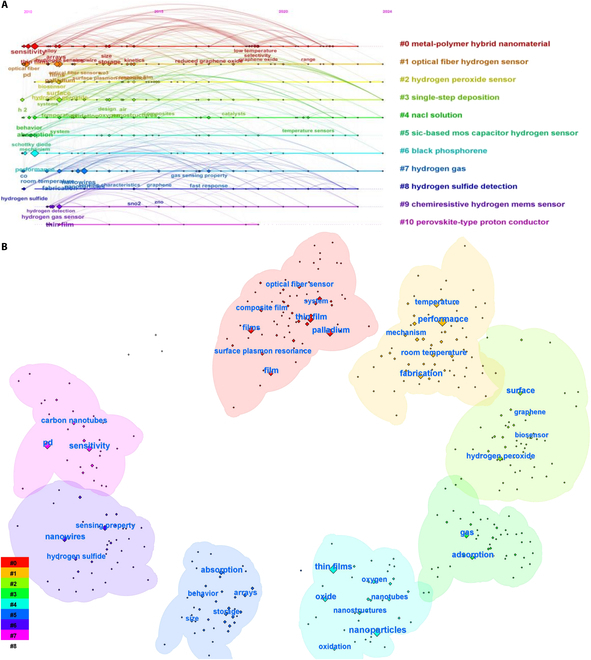
AIoT in hydrogen sensing. (A) Keyword clustering mapping. (B) Keyword co-occurrence analysis.

In order to accurately and clearly reveal the intrinsic connections, the overall trend of a discipline from a holistic perspective to a comprehensive literature dataset was discussed. The web of science core collection was used as the database source, and the search period spanned from 2010 to 2024. A total of 1,024 articles were obtained for keyword analysis. Meanwhile, CiteSpace’s co-word analysis feature was also adopted on keyword clustering maps. As shown in Fig. 3A, the main research contents of the clusters are as follows: metal-polymer hybrid nanomaterial, optical fiber hydrogen sensor, redox-responsive photonic crystal, single-step deposition, Pt material, conducting polymer nanoparticle, etc. [24,25]. Using CiteSpace’s burst detection feature, as shown in Fig. 3B, it provides a dynamic and comprehensive perspective, making it more suitable for tracking research frontiers than high-frequency keywords. Burst detection of keywords from cited references published between 2010 and the present yielded several burst keywords, which contains nanomaterial structures, optical sensor mechanisms and modes, and fabrication–absorption mechanisms. Therefore, finding efficient and cost-effective methods for fabricating hydrogen sensors is crucial for their industrialization and continuous improvement of both selectivity and sensitivity.

## Nanomaterials and Hybrid Structures for Sensitivity

**Fig. 4.**
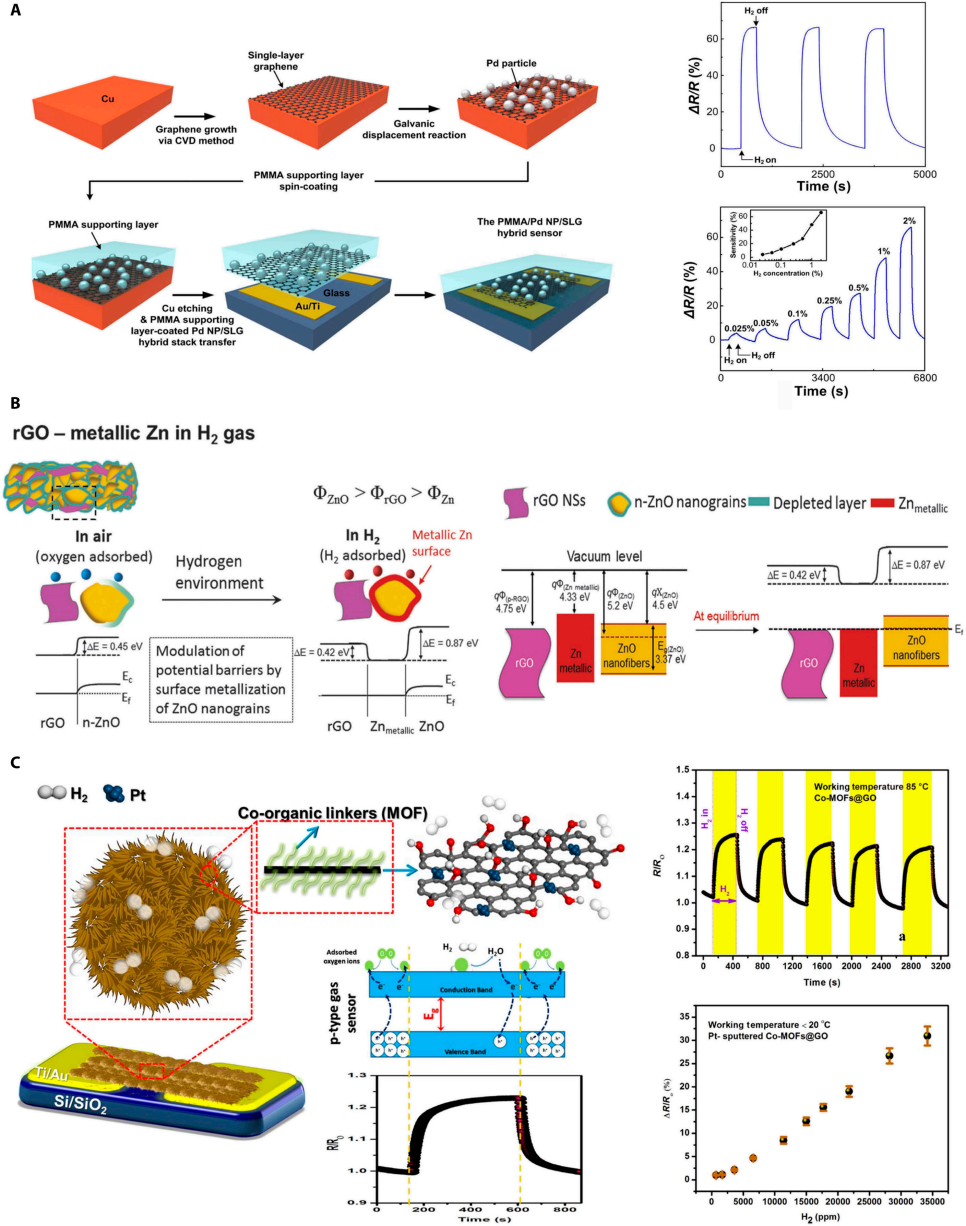
Hydrogen sensing with composite materials. (A) PMMA membrane-coated Pd nanoparticle/single-layer graphene hybrid sensor [32]. (B) rGO-loaded ZnO nanofiber sensor [33]. (C) Nanocomposite GO/metal organic framework sensor [34].

To enhance the sensitivity and selectivity of hydrogen sensors, researchers have turned their focus to nanomaterials and hybrid structures [25–28]. With innovative designs and the unique properties of nanomaterials, more efficient, accurate, and reliable detection can be achieved. 2D materials, such as graphene, characterized by its single-atom-thick honeycomb lattice of carbon atoms, offer an extensive surface-to-volume ratio, rendering it highly suitable for ultra-sensitive detection applications. Researchers have innovatively integrated hydrogen-sensitive metal oxide nanoparticles with monolayer graphene at the nanoscale, enhancing electron mobility within the sensors [29–31]. As shown in Fig. 4A, Hong et al. [32] designed a sensor wherein Pd nanoparticles were deposited onto single-layer graphene via an electro-displacement reaction. A novel polymer film coating was applied to allow hydrogen to permeate in detection. The interaction between the absorbed hydrogen and the Pd nanoparticles enabled a quantifiable method for measuring hydrogen concentration. Additionally, the integration of reduced graphene oxide (rGO) with zinc oxide (ZnO) nanofibers represents an important advance in the sensitivity. The Pd/rGO sensor may experience a decrease in sensitivity at temperatures above 150 °C due to graphene oxidation and Pd agglomeration, but SiO₂ encapsulation allows for operation up to 200 °C. As shown in Fig. 4B, Abideen et al. [33] leveraged the synergistic effects of the high surface area of rGO and the unique properties of ZnO nanofibers. The sensor demonstrates a highly sensitive response curve with a concentration of 10 ppm. The polymethyl methacrylate (PMMA) layer enhances resistance to humidity interference [stable at 30% to 70% relative humidity (RH)], but the mechanical flexibility has not yet been verified. Moreover, the development of hydrogen sensors based on graphene oxide (GO) hybrid MOFs represents an important advancement. As shown in Fig. 4C, Fardindoost et al. [34] combined the high surface area and adsorption capabilities of MOFs with the conductivity of GO. The inclusion of Pt as catalyst facilitates the dissociation of hydrogen molecules, which exhibits strong hydrogen sensing capabilities with excellent response and recovery times of 9 and 12 s, particularly hydrogen concentrations among 700 to 35,000 ppm. The sensor exhibits stable response to hydrogen within the temperature range of room temperature to 150 °C (detection limit of 10 ppm). However, the MOF porous structure is not suitable for high-pressure environments, and its long-term stability under higher temperatures (>200 °C) or corrosive environments has not been validated.

**Fig. 5.**
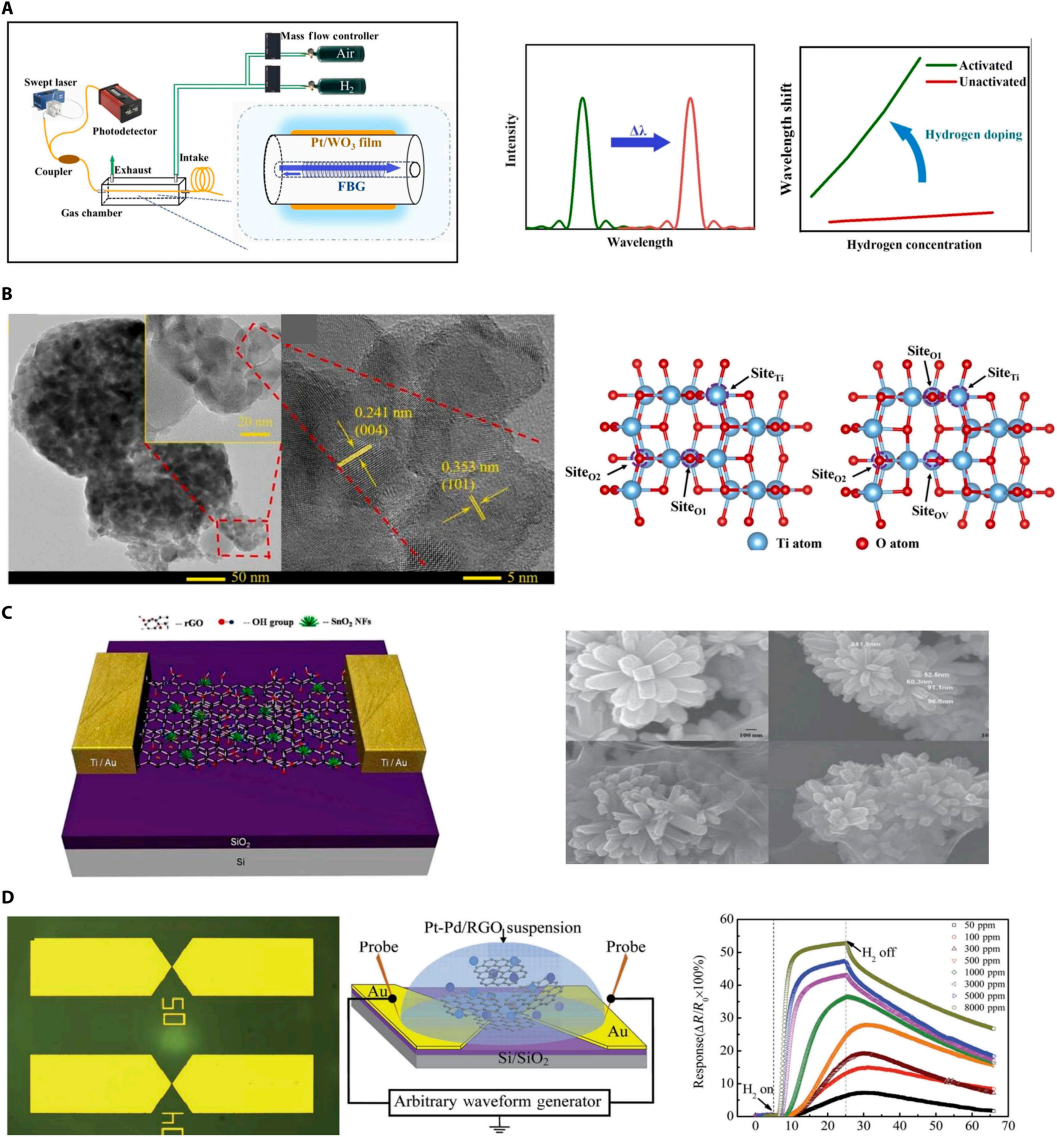
Hydrogen sensing with the aid of metal particles. (A) Hydrogen sensor based on hydrogen-doped Pt/WO_3_ [35]. (B) Metal–organic framework-derived oxygen-rich vacancies and porous TiO_2_ nanotablets for hydrogen detection [36]. (C) Hydrogen sensing using rGO and tin oxide nanoflowers [37]. (D) Pt-Pd/rGO-based sensor [38].

As shown in Fig. 5A, Wang et al. [35] developed a highly sensitive fiber Bragg grating (FBG) hydrogen sensor based on hydrogen-doped Pt/WO_3_. They pretreated the Pt/WO_3_ material to achieve a detection limit as low as 30 ppm. Additionally, by measuring the wavelength difference of the FBG pairs, they eliminated the influence of environmental temperature fluctuations. Meanwhile, as shown in Fig. 5B, Zhang et al. [36] achieved high-response hydrogen sensing using oxygen-rich vacancies and porous TiO_2_ nanosheets derived from MOFs. This sensor demonstrates excellent gas-sensitive performance at room temperature, exhibiting rapid response times, long-term stability, and selectivity across a wide concentration range of 25 to 4,000 ppm. Furthermore, as shown in Fig. 5C, Venkatesan et al. [37] utilized the synergistic properties of rGO and tin oxide nanofibers. The sensor showed important enhancement in sensing performance, with a response of 19.6% to 200 ppm hydrogen. As shown in Fig. 5D, Peng et al. [38] developed a sensor based on Pt-Pd/rGO. The sensor demonstrated stable sensing performance across varying hydrogen concentrations (50 to 8,000 ppm) with a quick recovery due to physical desorption. However, the sensor’s sensitivity decreased under high humidity and temperatures, limiting performance stability in diverse environmental conditions. At the same time, the aforementioned sensors are not suitable for extreme situation detection.

In the development of high-performance hydrogen gas sensors, nanocomposite hybridization techniques play a crucial role in enhancing sensitivity, selectivity, response speed, and stability. Various synthesis strategies—ranging from simple physical mixing to precise atomic layer deposition—allow for the construction of nanostructured sensing materials with tailored electronic and catalytic properties. However, each method presents its own set of advantages and limitations in terms of process complexity, scalability, interfacial bonding, and applicability to different sensing scenarios. Table 1 provides a systematic comparison of commonly used nanocomposite hybridization methods for hydrogen gas sensors, covering aspects such as synthesis approach, typical materials, key advantages, and inherent limitations.

**Table 1.** Comparison of different nanohybrid methods

Hybridization method	Material combination strategy	Typical materials	Advantages	Limitations
Physical mixing	Direct mixing of 2 or more nanomaterials without strong interfacial reactions	SnO_2_ + Pd nanoparticles	Simple process, low cost, scalable; multiphase synergy enhances sensing performance	Nonuniform distribution, weak interfacial bonding, limited stability
Sol–gel method	Homogeneous hybridization in solution phase followed by solidification	ZnO + Pt nanoparticles	Uniform composition, controllable structure, suitable for porous materials	Long processing time, sensitive to parameter control
Hydrothermal/solvothermal method	Nanostructures grown in situ under high temperature and pressure	TiO_2_@Pd, MoS_2_@Pt	Good crystallinity, strong material adhesion, stable interface	Strict temperature/time control, low throughput, limited scalability
In situ deposition	Direct growth of functional materials on sensing substrates	In_2_O_3_@Au, WO_3_@Pd	Tight interfacial bonding, enhanced electronic coupling, fast response	Equipment-intensive, mainly for research-level work
Atomic layer deposition	Atomically precise control of material layer thickness	ALD-Pt/ZnO	High precision, excellent interface quality, reproducible	Expensive equipment, complex process, time-consuming
Electrochemical deposition	Electrochemical control of deposition location and morphology	Pd@rGO, NiO@CNTs	Low cost, mild conditions, localized structure control	Adhesion and thickness control can be challenging
Gas phase deposition (CVD/PVD)	Deposition of nanostructures from vapor-phase precursors	CNT/Pd, graphene/Pt	Uniform hybridization, adjustable structure, high reproducibility	High energy consumption, requires high-temperature conditions and compatible substrates

## Optical Effects for Hydrogen Sensing

Optical hydrogen sensors offer a promising solution for reliable, sensitive, and real-time hydrogen detection. These sensors leverage various optical principles to detect hydrogen concentrations with high accuracy and response speed, often outperforming conventional sensors in specific aspects. The 4 main types includes fiber-optic, surface plasmon resonance (SPR), colorimetric, and photoacoustic hydrogen sensors. For most optical hydrogen sensors, they are suitable for a wide range of high- and low-temperature detection conditions. However, it is important to be aware of hydrogen embrittlement failure under prolonged high temperatures. Additionally, while most optical sensors can be used in high-pressure environments, attention must be paid to the structural collapse of the nanoscale porous hydrogen-sensitive materials (such as MOFs) under high pressure. Most research-oriented optical hydrogen sensors have not yet considered corrosive conditions, which can be addressed in subsequent commercial applications by using coatings or films to enhance corrosion resistance.

### Fiber-optic hydrogen sensors

**Fig. 6.**
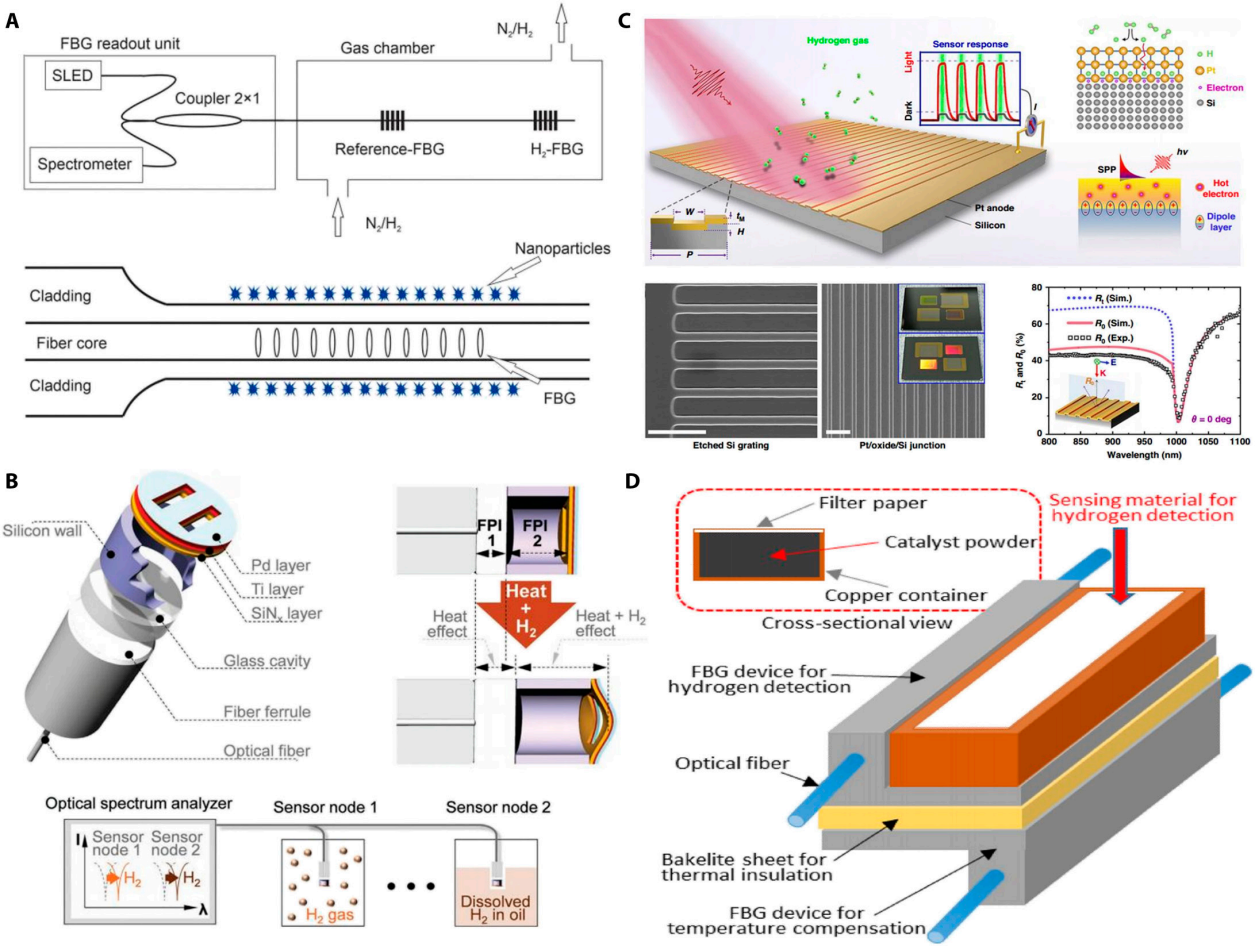
Optical reflection sensing. (A) Optical fiber evanescent hydrogen sensor based on Pd nanoparticles [47]. (B) Cascaded interferometers incorporated optical fibers [48]. (C) Plasmon-induced hot electron–molecule interaction [49]. (D) Catalytic combustion-type optical FBG hydrogen gas sensor using Pt-loaded fumed silica powder [50].

The fiber-optic hydrogen sensor is based on 2 types of sensitive films: Pd-based films [39] and tungsten oxide (WO_3_) [40], coated at the tip of the fiber or along the length of the fiber. By depositing the Pd film onto the surface of the optical fiber, changes in hydrogen concentration can alter the volume and dielectric constant of the Pd, thereby inducing variations in the optical signal in terms of intensity, wavelength, and/or phase [19,20,41–46]. When these sensitive films are coated on the surface of an optical fiber, the optical signal varies with hydrogen concentration. As shown in Fig. 6A, Abdalwareth et al. [47] developed a hydrogen sensor based on the evaluation of the reflection signal intensity from an FBG. This sensor employs an etched single-mode fiber to enhance the interaction of the evanescent field within the sensing region, with Pd nanoparticles coated on its surface. The dynamic operating range of this sensor is from 0.3% to 5.0%, making it suitable for early-warning application to ensure safety. As shown in Fig. 6B, Lee et al. [48] proposed a fiber-optic hydrogen sensor based on a Fabry–Pérot interferometric structure. This sensor utilizes the volumetric expansion of Pd upon hydrogen exposure to adjust the resonance mode of the interferometer cavity, achieved by altering the shape of a silicon nitride mirror from planar to convex with a detection limit of 15 ppm. In Fig. 6C, Wen et al. [49] introduced an on-chip plasmonic-catalyzed hydrogen sensor based on a metal–insulator–semiconductor nanojunction. This device enhances the sensing signal through an interfacial dipole charge layer and plasmonic hot electron-modulated photoresponse, achieving a detection limit as low as 1 ppm. Finally, as shown in Fig. 6D, Okazaki et al. [50] developed a hydrogen sensing device based on FBG, which utilizes the temperature change induced by the catalytic reaction of hydrogen on Pt-loaded silicon catalyst powder. The detection limit is approximately 0.2%, and the device exhibits excellent long-term stability.

**Fig. 7.**
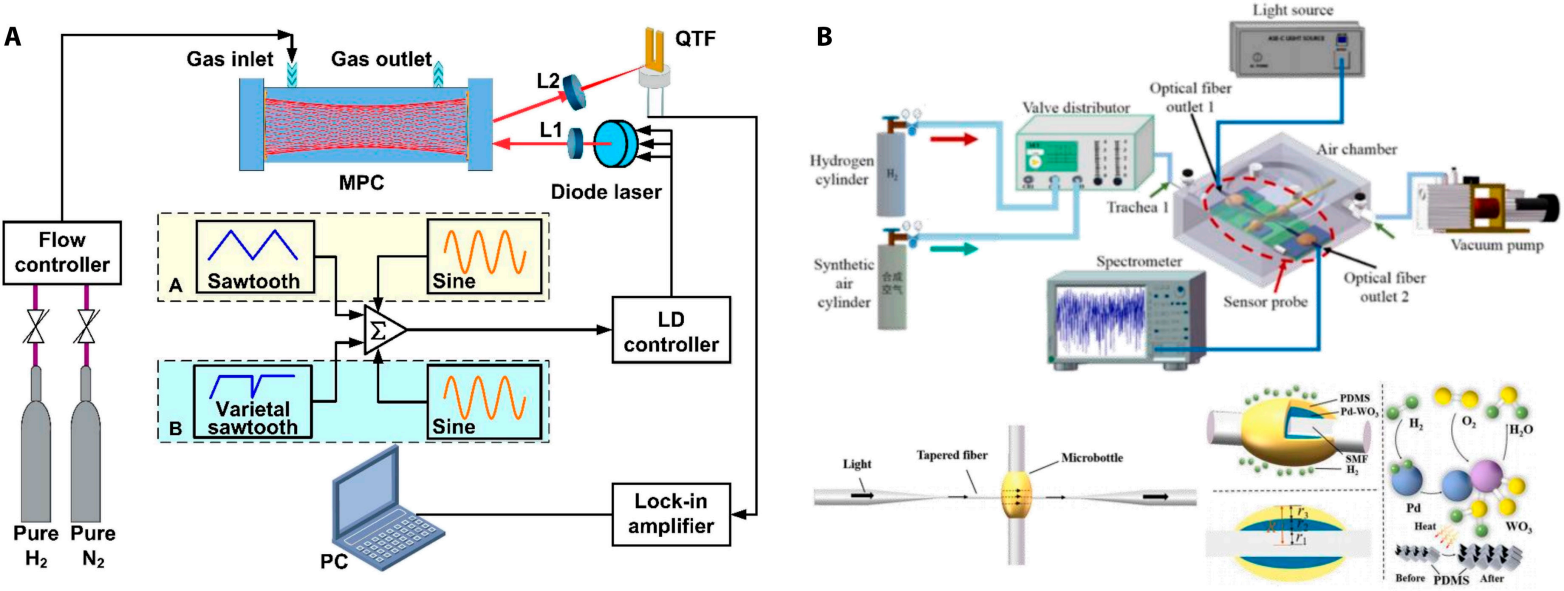
Light-induced optical sensors. (A) Hydrogen detection based on LITES [51]. (B) Optical fiber hydrogen sensor based on self-assembled PDMS/Pd-WO_3_ microbottle resonator [52].

As shown in Fig. 7A, Ma et al. [51] demonstrated a high-sensitivity hydrogen sensor based on light-induced thermoelastic spectroscopy (LITES). The sensor employs a shallow neural network fitting algorithm for signal denoising, achieving an excellent linear response to varying hydrogen concentrations with a minimum detection limit of 45 ppm. As shown in Fig. 7B, Zhang et al. [52] reported a fiber-optic hydrogen sensor based on a self-assembled microbottle resonator. Hydrogen molecules diffuse through the polydimethylsiloxane (PDMS) and react with Pd-WO_3_, leading to a shift in the resonant wavelength of the microbottle resonator due to the heat absorption of reaction by PDMS. This sensor achieves hydrogen detection through wavelength shift, demonstrating good resistance to temperature and humidity interference.

### SPR hydrogen sensors

**Fig. 8.**
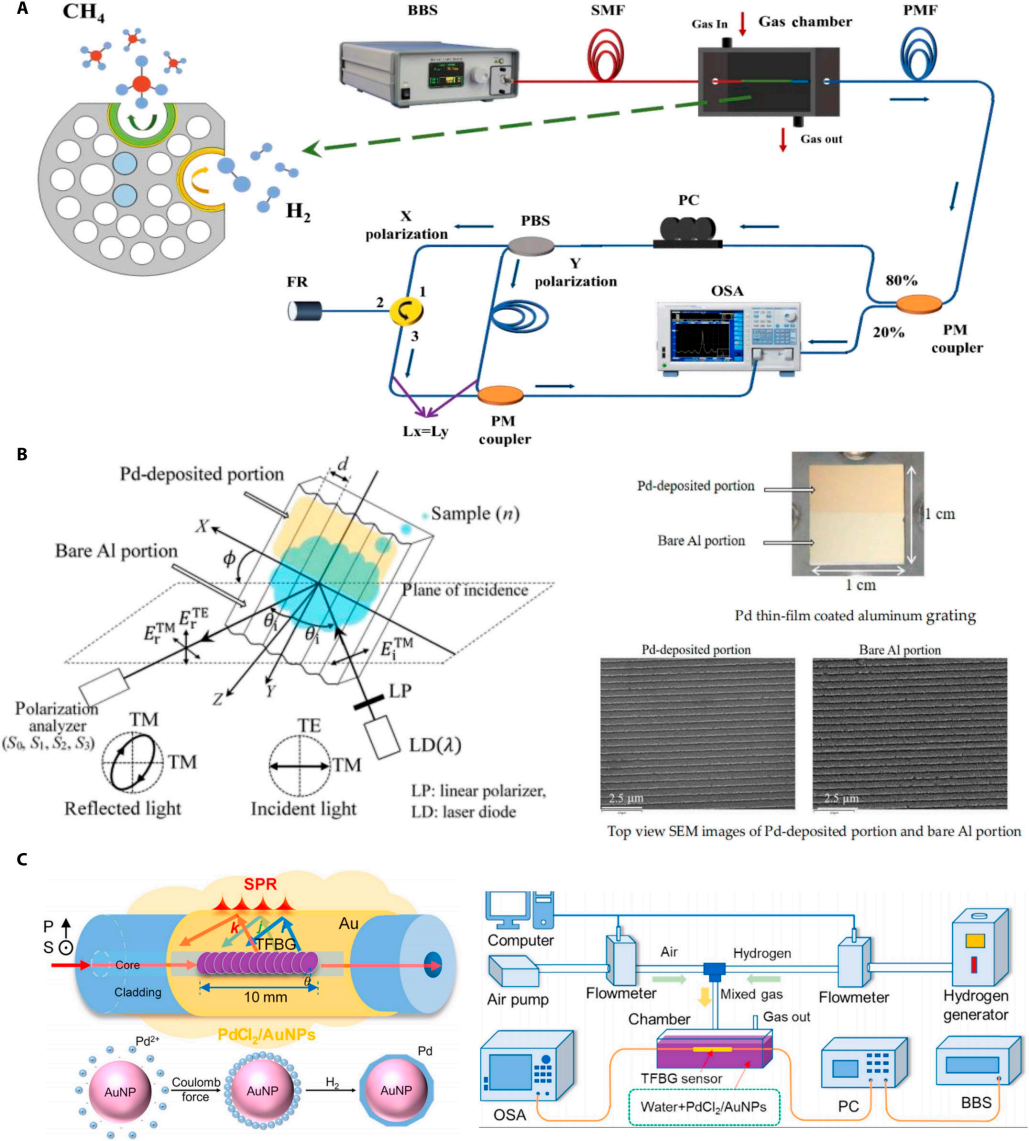
SPR hydrogen sensors. (A) Anchor-shaped photonic crystal fiber [53]. (B) Polarization property in Pd [54]. (C) Optical fiber hydrogen detection in liquid environment [55].

SPR hydrogen sensors leverage an optical phenomenon to achieve highly sensitive detection. SPR occurs when polarized light hits a metal surface at a specific angle, exciting free electrons and creating oscillations known as surface plasmons. As shown in Fig. 8A, Xiao et al. [53] designed a novel high-sensitivity multi-parameter sensor based on SPR in an anchor-shaped photonic crystal fiber, aimed at simultaneous detection of hydrogen, methane, and temperature with built-in temperature self-compensation. As shown in Fig. 8B, Matsuda et al. [54] developed an innovative sensor based on the polarization properties of light, utilizing the SPR effect on a Pd thin-film-coated aluminum grating. The sensor detects hydrogen gas by monitoring rapid variations in the normalized stokes parameter around the resonance angle. The shifts in the Pd film’s refractive index enabled sensitive detection at concentrations close to the lower explosive limit. Furthermore, Almeida et al. [46] introduced an optical fiber hydrogen sensor leveraging SPR to enhance sensitivity and enable remote monitoring in harsh environments. However, its performance is limited by the high optical absorption of Pd, which negatively affects both sensitivity and response time. As shown in Fig. 8C, Xie et al. [55] proposed a method for detecting hydrogen in liquid environments using a gold-plated tilted FBG (TFBG) and a PdCl₂/AuNPs solution. This method detects hydrogen by exciting the SPR of the gold-plated TFBG. The sensor achieves a sensitivity of up to 0.99 dB/% in liquid environments.

### Colorimetric hydrogen sensors

**Fig. 9.**
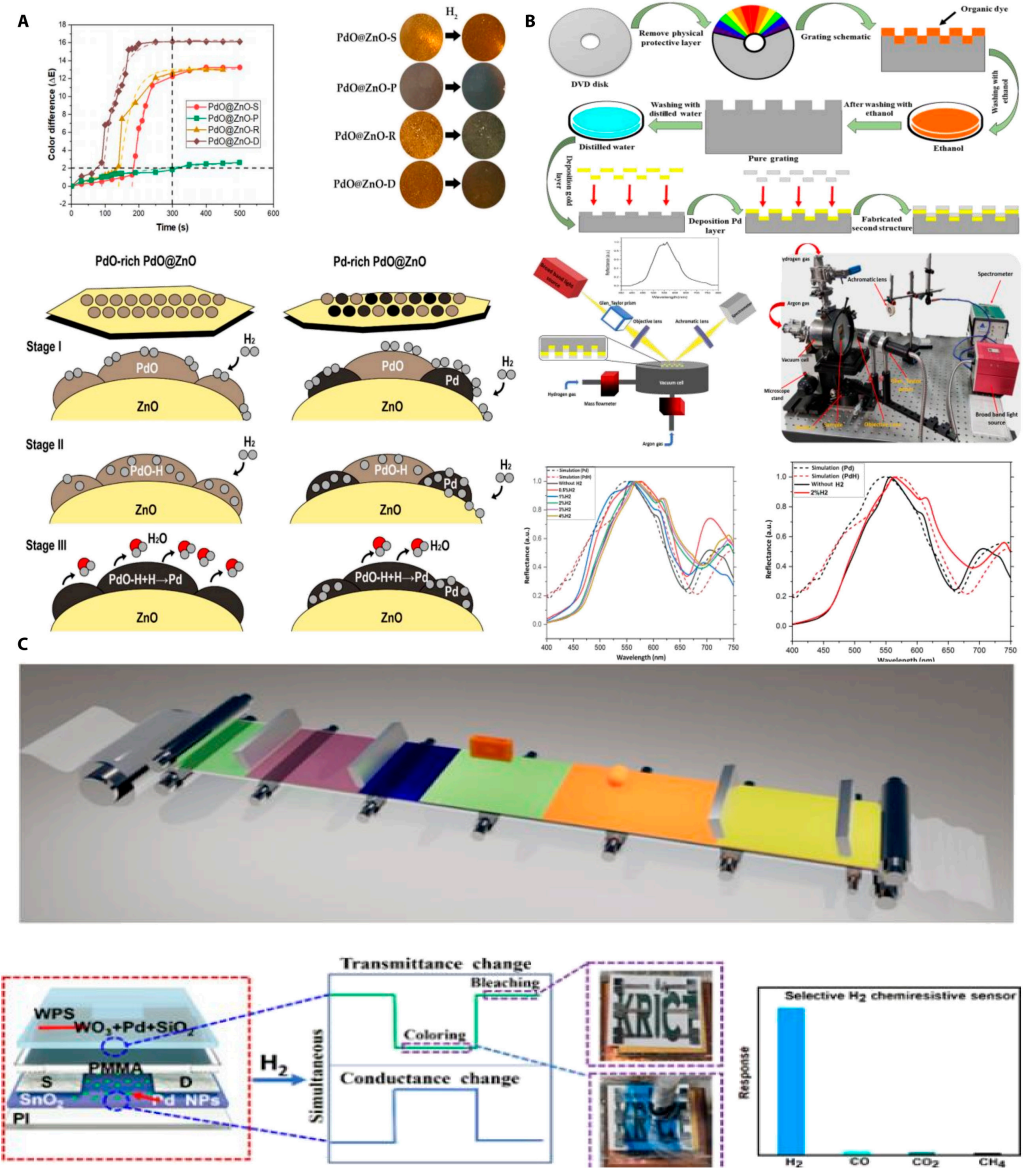
Colorimetric hydrogen sensors. (A) Colorimetric hydrogen sensors of PdO@ZnO hybrids [56]. (B) Colorimetric plasmonic hydrogen sensor based on 1D nano-gratings [57]. (C) Large-area printed oxide film sensors [58].

Colorimetric hydrogen sensors operate based on a simple visual detection mechanism that relies on color changes induced by chemical reactions between hydrogen and a reactive material. These sensors typically consist of a substrate coated with a Pd-based compound that undergoes a visible color shift upon hydrogen exposure. As shown in Fig. 9A, Hong et al. [56] developed a PdO-decorated ZnO (PdO@ZnO) colorimetric hydrogen sensor, representing an important advancement in hydrogen detection and offering visual detection without external power sources. However, the sensor’s sensitivity is reduced in ambient air due to oxygen interference, which diminishes the observed color change. As shown in Fig. 9B, Zarei et al. [57] presented an innovative plasmonic hydrogen gas sensor, which detects hydrogen by monitoring changes in the color and resonance wavelength of the Pd layer. Despite the enhanced sensitivity, the sensor suffers from instability due to cyclic expansion of the Pd lattice, a narrow pressure range typical of single-metal systems, and limited imaging resolution for detecting hydrogen at ultra-low concentrations. As shown in Fig. 9C, Girma et al. [58] presented an innovative hydrogen sensor based on polymer–noble metal–metal oxide films, specifically utilizing spin-coating and printing techniques. Despite its effectiveness, the sensor faces limitations in varying humidity conditions and slight temperature dependence.

### Photoacoustic hydrogen sensors

**Fig. 10.**
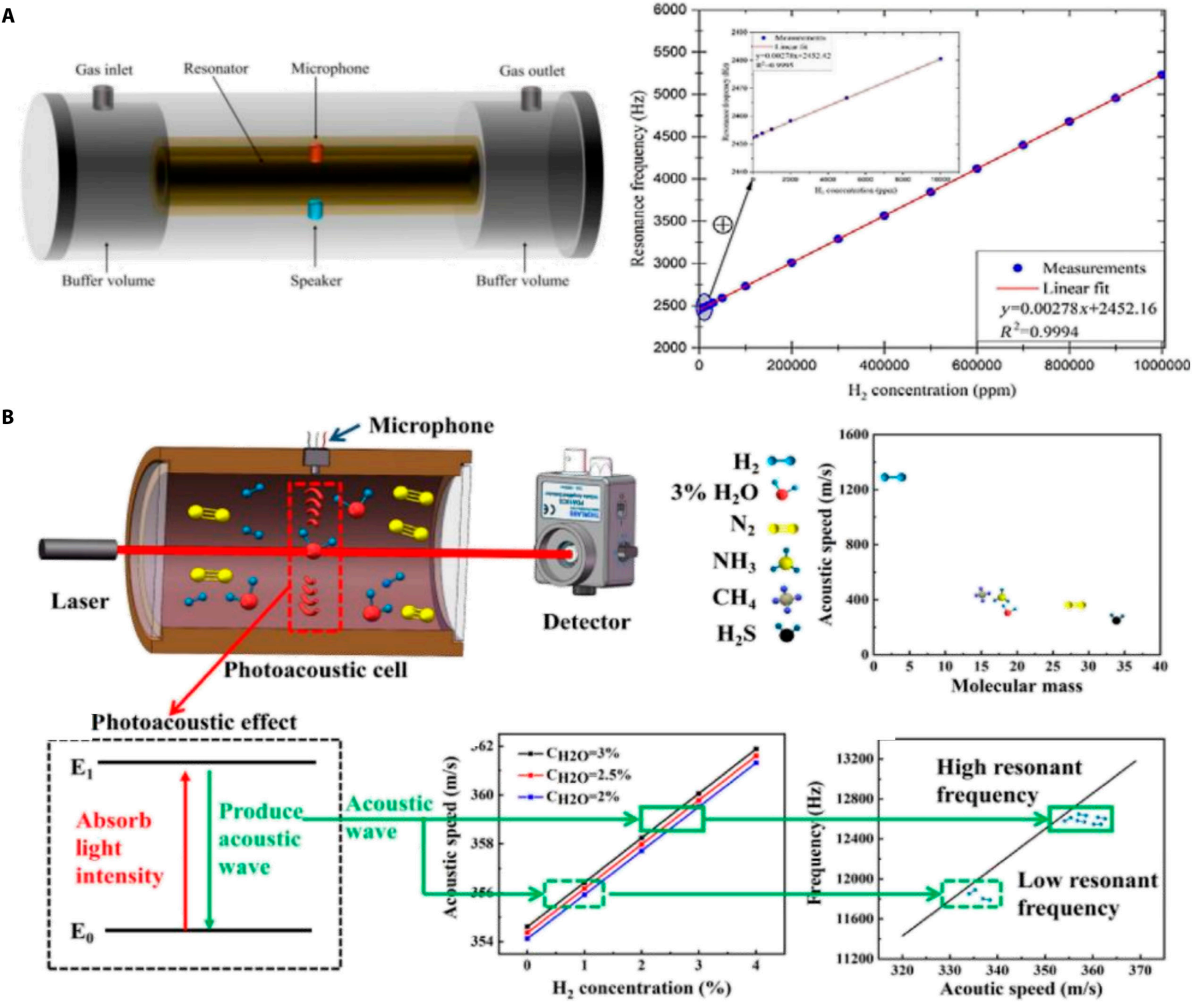
Photoacoustic hydrogen sensors. (A) Resonant photoacoustic cell for hydrogen gas detection [59]. (B) Photoacoustic dual-gas sensor [60].

Photoacoustic hydrogen sensors operate using photoacoustic spectroscopy, which combines optical and acoustic principles to measure gas concentrations. Hydrogen molecules are exposed to a modulated light source, usually in the infrared range, that excites the molecules and causes them to release heat as they return to a relaxed state. As shown in Fig. 10A, Wang et al. [59] proposed a hydrogen sensor using resonance frequency tracking in a resonant photoacoustic cell, enabling stable and precise hydrogen detection. The sensor achieves a minimum detection limit of approximately 74 ppm, with a response time of about 5 s at atmospheric pressure. In Fig. 10B, Ye et al. [60] presented important innovation in hydrogen and water vapor sensor by utilizing a dual-gas detection system based on photoacoustic spectroscopy with an optimized H-type photoacoustic cell. The system includes a minimal detection limit of 138.69 ppm for hydrogen and 3.70 ppm for H_2_O, a response time of 0.51 s, and a recovery time of 0.38 s.

## Move to Next-Generation Hybrid Functional Mechanisms

**Fig. 11.**
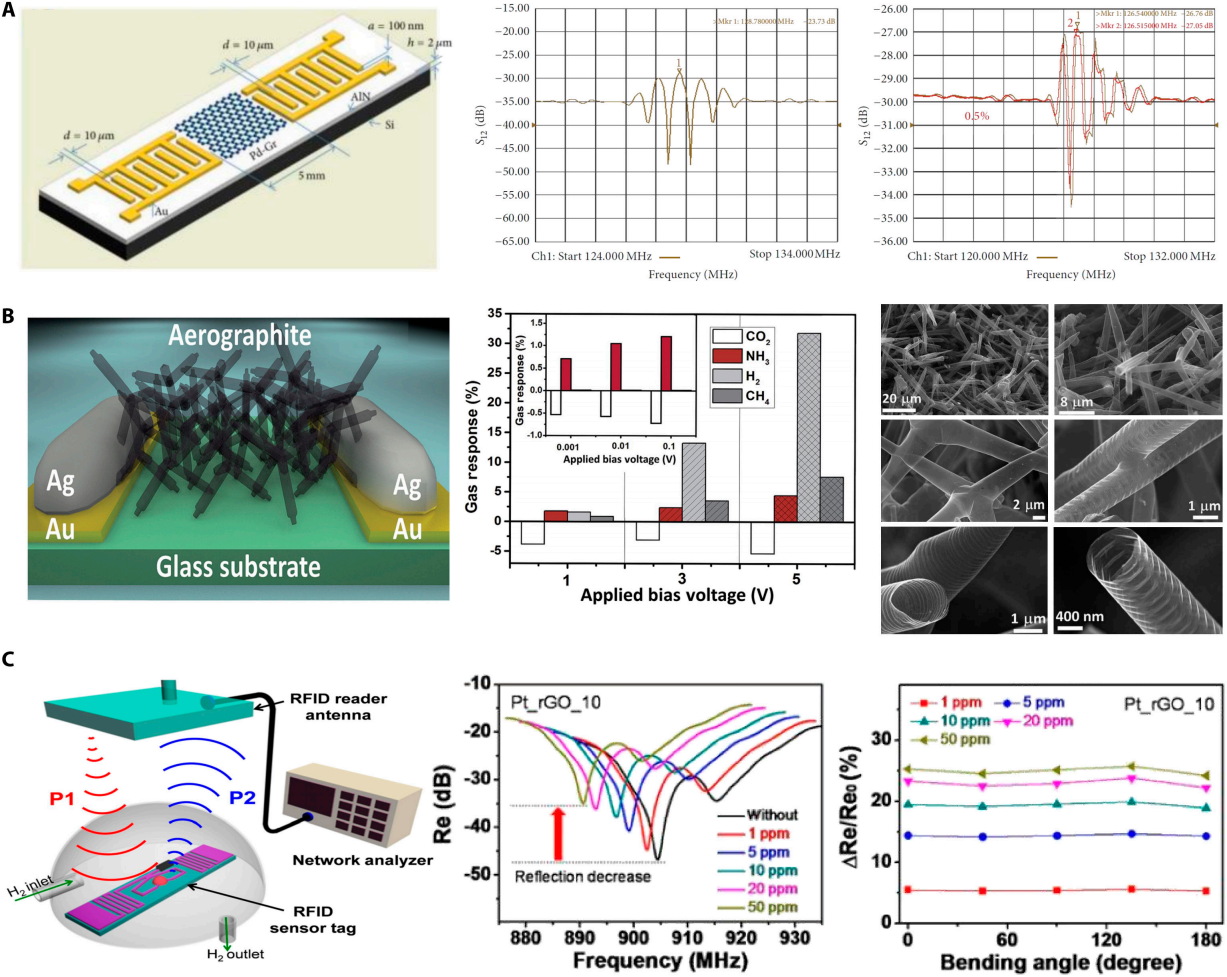
Novel detection mechanisms of hydrogen sensors. (A) Hydrogen gas sensing using Pd–graphene nanocomposite material based on SAW [61]. (B) Ultra-light aerographite sensor [63]. (C) Wireless hydrogen sensor based on Pt/graphene-immobilized radio-frequency identification tag [64].

Beyond innovations in nano-scale hybrid structures and optical sensors, researchers have endeavored to apply hybrid functional mechanisms to hydrogen sensors, aiming to meet the demands for high sensitivity and compatibility in detecting low concentrations [61]. As shown in Fig. 11A, Ha et al. [62] deposited Pd–Gr nanocomposite materials on a SAW device, which consists of interdigitated transducers on an aluminum nitride substrate. The presence of hydrogen induces changes in the mass and conductivity of the Pd–Gr nanocomposite material, affecting the velocity and attenuation of the SAWs. The sensor has not been tested under extreme temperature or high-pressure conditions, and it is speculated that its high-temperature performance (>80 °C) is limited by Pd hydrogen embrittlement, while the SAW substrate may affect the consistency of sensitivity due to temperature drift. As shown in Fig. 11B, aerographite sensors represent a breakthrough in low-power, tunable, and ultralight gas sensor design, consisting of a 3D network of graphene and nano-aerographite [63]. They combine the conductivity and high surface area of their constituent materials to create sensor with exceptional sensitivity and selectivity. The ultra-lightweight aerogel graphene sensor operates within an ultra-wide temperature range of −196 °C to 300 °C (detection limit of 1 ppm). Its lightweight porous structure is capable of withstanding low to medium pressures (less than 5 bar), making it suitable for aerospace applications. As shown in Fig. 11C, Lee et al. [64] developed a wireless hydrogen smart sensor based on Pt-decorated rGO affixed to radio-frequency identification (RFID) tags, representing an important advancement in wide-area wireless hydrogen monitoring technology. This results in detectable changes in the RFID tag’s signal, which can be remotely monitored through a network analyzer connected to an RFID reader and antenna. The flexible substrate of the sensor is adaptable to mechanical deformation; however, extreme temperatures (above 80 °C or below −20 °C) and high-pressure scenarios have not been verified. High temperatures may impact the stability of the RFID chip circuitry.

**Fig. 12.**
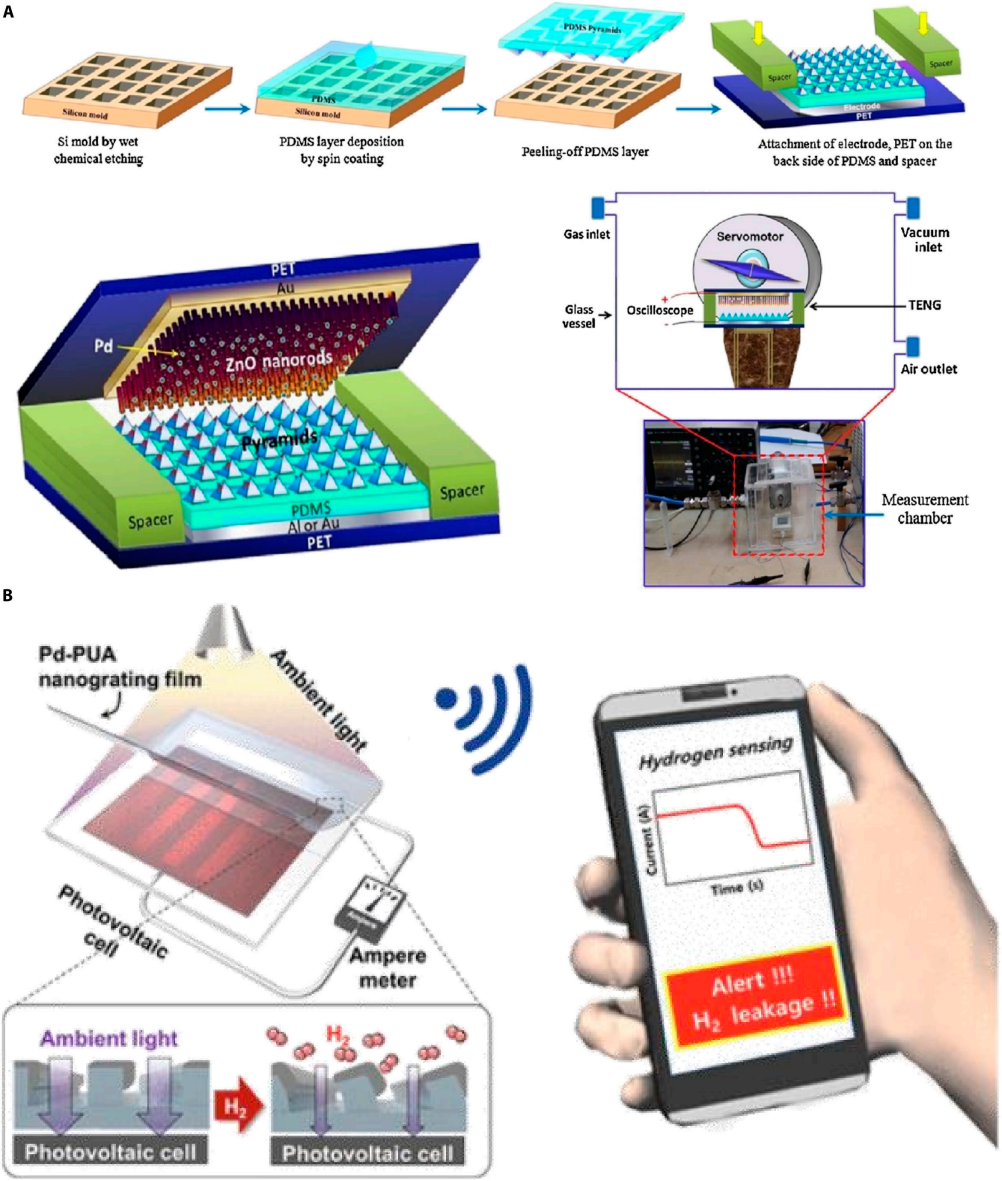
Two type of self-powered hydrogen sensors. (A) Self-powered active hydrogen gas sensor based on triboelectric effect [68]. (B) Pd-polymer nanograting film for self-powered hydrogen gas sensor [69].

Another groundbreaking application in hydrogen sensor technology is the exploration of self-powered sensors utilizing the triboelectric effect. These sensors represent a paradigm shift in energy requirements for gas detection, operating without an external power source [65–67]. The mechanism employs a surface-modified PDMS film adorned with micropyramids to enhance its surface area. As shown in Fig. 12A, Uddin and Chung [68] developed a hydrogen sensor that harnesses the triboelectric effect for detection. Upon exposure to hydrogen gas, the electrical properties of the Pd nanoparticles change, modulating the electrical output generated by the triboelectric effect. This modulation is directly correlated with the concentration of hydrogen gas, providing a basis for detection. The sensor maintains a stable output within the temperature range of −20 to 35 °C through a porous structure and heterojunction design, and its durability has been validated through 40,000 mechanical cycle tests. However, its application and detection in high-temperature environments may be affected. Furthermore, as shown in Fig. 12B, Seo et al. [69] proposed a self-sufficient, reliable hydrogen sensor that ingeniously integrates chemo-mechanically active nanostructured films with photovoltaic cells for autonomous operation. Notably, this mechanism allows for the detection of wide range of hydrogen concentrations (0.1 to 4.0%) by simply measuring the self-generated current of the photovoltaic cell. The sensor responds quickly at room temperature (<1 s), but its performance under extreme temperatures or humidity has not been explicitly tested. Nevertheless, its core materials, such as Pd-based composites, may face challenges like hydrogen embrittlement at high temperatures or reduced activity at low temperatures.

## Move to AIoT System

**Fig. 13.**
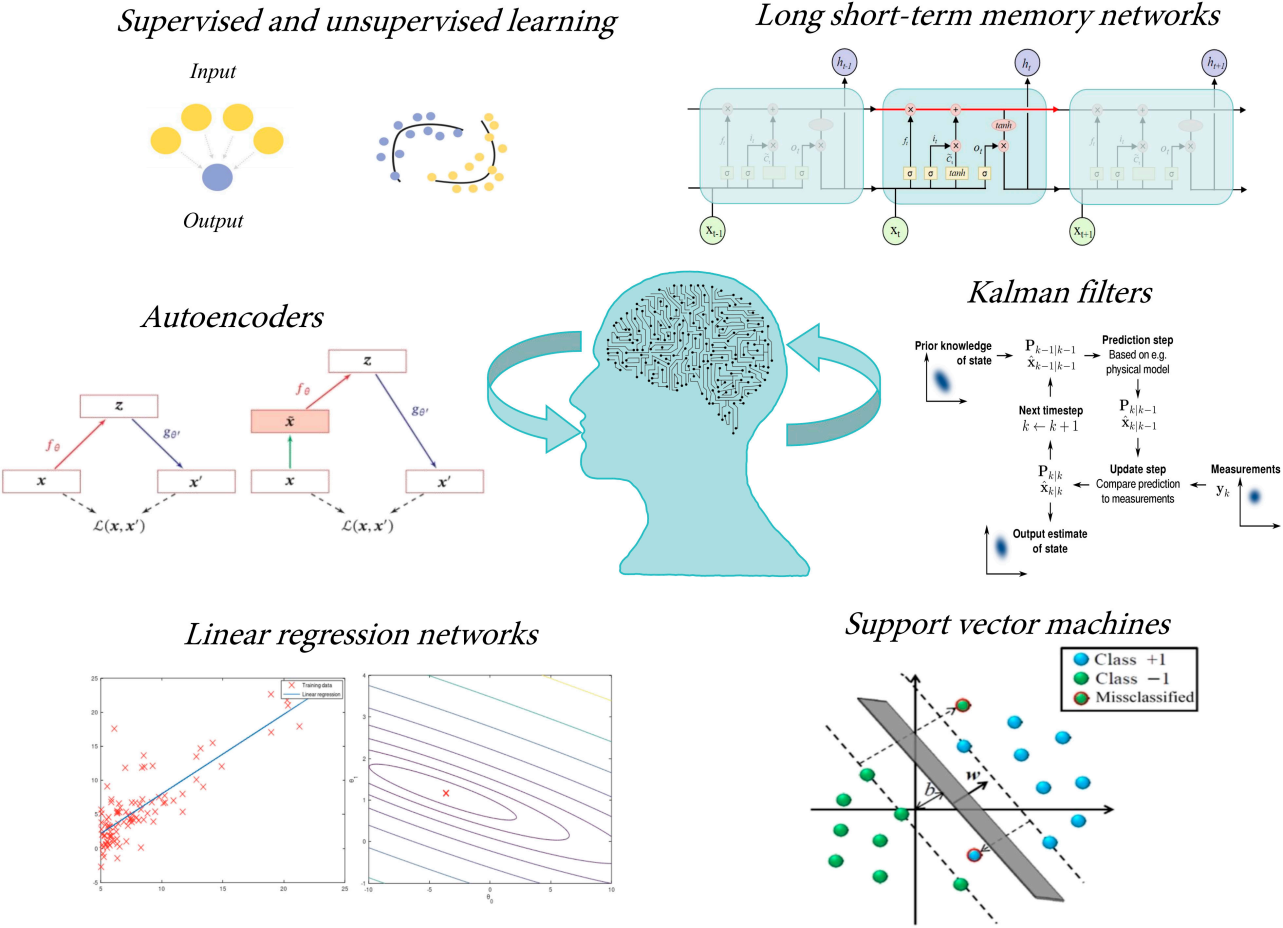
Deep learning architectures for hydrogen sensor signals.

The aforementioned sensor configurations demonstrate high precision and reliability in hydrogen detection through the application of novel sensing mechanisms and advanced materials. To further enhance sensor functionality, the development of artificial intelligence offers new perspectives for signal processing and feature extraction, as shown in Fig. 13 [70–72]. By converting environmental parameters monitored by the sensor into electrical signals and processing these data, ML techniques can extract rich and complex features embedded in the signal [73–75]. For instance, using gated recurrent unit neural network-based ML methods, it is now possible to extract information about individual components from overlapping signals of a single gas sensor without the need for pre-separation, thus enabling rapid and straightforward detection of mixture components. Principal components analysis (PCA) employs orthogonal transformation to convert a set of potentially correlated variables into linearly uncorrelated principal components, thus reducing dimensionality and revealing intrinsic data patterns. Firstly, sensors can be influenced by environmental factors during operation, leading to signal drift or bias. Through supervised and unsupervised learning, ML algorithms can calibrate sensors, providing more accurate measurements [76–80]. Deep learning (DL) methods are particularly effective in handling complex and noisy signals. Utilizing CNNs and autoencoders, DL can efficiently denoise signals and improve overall sensing performance [81–85]. Regression models such as linear regression and deep neural networks (DNNs) can accurately predict hydrogen concentrations in the environment [86–89]. Furthermore, anomaly detection algorithms and recurrent neural networks (RNNs), particularly long short-term memory networks (LSTMs), can be employed for real-time monitoring of sensor signals, detecting and diagnosing faults to ensure the system’s normal operation [90–95]. By leveraging data fusion techniques like Kalman filters and ensemble learning methods, multiple sensor data can be effectively integrated to yield more reliable results [96–100]. For real-time monitoring and prediction, deep reinforcement learning methods such as support vector machines (SVMs) can achieve real-time processing and analysis of sensor data, ensuring system safety [101–104].

**Fig. 14.**
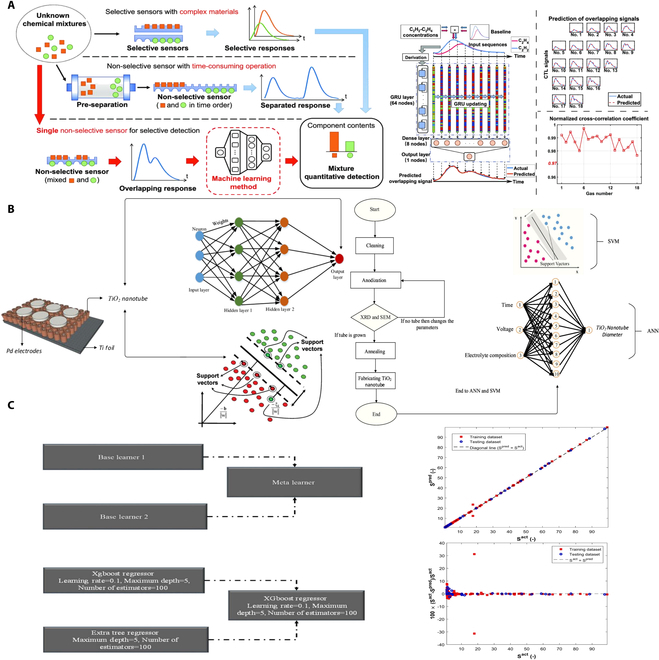
AIoT enhanced hydrogen sensing. (A) Selective detection of mixtures via single nonselective sensor [105]. (B) TiO_2_ nanotubes by electrochemical anodization and ML method for hydrogen sensors [106]. (C) Stacked ML model to compute the ZnO sensors [107].

Highly selective and sensitive detection of chemical mixtures via a single sensor is invariably affected by overlapping response signals. As shown in Fig. 14A, Liu et al. [105] employed a smart RNN architecture equipped with gated recurrent units (GRUs) to directly predict mixture compositions from overlapping signals, achieving prediction accuracies of 100% for known samples and 92.22% for unknown samples. The integration of RNN into selective sensing offers a novel and efficient approach for selective quantitative detection through a single nonselective sensor. As shown in Fig. 14B, Isik et al. [106] studied the preparation methods of TiO₂ nanotubes using different anodization parameters and applied them to performance testing of hydrogen sensors. The research focused on the simulation and prediction of nanotube diameter using ML methods, particularly SVM and artificial neural networks (ANNs), with some diameters completely estimated by models and not confirmed through experiments. This study further demonstrates that the combination of anodization experiments with ML models can effectively enhance the optimization efficiency of nanostructures, providing intelligent tools for sensor design. As shown in Fig. 14C, Vaferi et al. [107] proposed a regression model to accurately correlate the sensitivity of ZnO-based sensors with the chemical composition of nanocomposites, hydrogen concentration, and temperature by extreme gradient boosting regressors. The precision of this model surpasses that of traditional artificial neural networks, with an average absolute error (MAE) of 0.11, mean squared error (MSE) of 0.31, and mean absolute percentage error (MAPE) of 1.14% based on 208 actual sensor sensitivity readings.

**Fig. 15.**
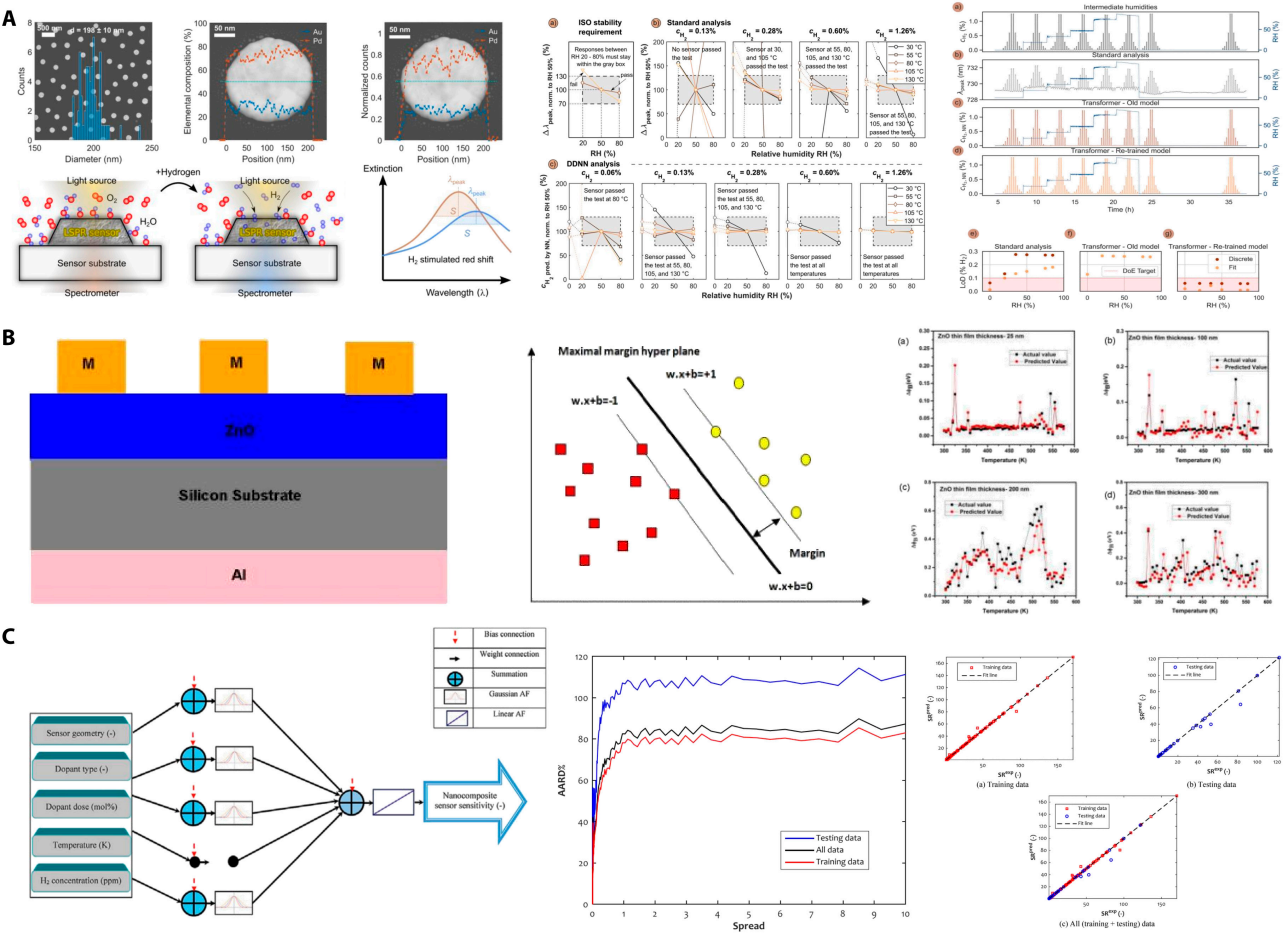
Artificial intelligence enhanced hydrogen sensors. (A) Neural network enabled nanoplasmonic hydrogen sensors [108]. (B) Thin-film hydrogen sensor based on macro-model [109]. (C) Hydrogen sensors based on general regression artificial neural network [110].

In addition, as shown in Fig. 15A, Tomeček et al. [108] utilized neural network algorithms to improve the LoD of Pd-based hydrogen sensors under high humidity conditions. Initially, a deep dual neural network (DDNN) was implemented to analyze the complete extinction spectrum measured by the sensors. Subsequently, the transformer algorithm was used to better handle complex long-time series data, eliminating noise and ambiguous signals, and accurately detecting hydrogen concentrations as low as 0.01% under various humidity conditions. As shown in Fig. 15B, Rajan et al. [109] applied a least squares SVM (LS-SVM) to develop a regression model for predicting the output behavior of ZnO thin-film Schottky diode-based hydrogen sensors. The proposed modeling scheme provides guidance for the fabrication of ZnO thin-film Schottky diodes for hydrogen sensing applications. Finally, as shown in Fig. 15C, Zhong and Abdollahi [110] investigated the use of generalized regression (GR) neural networks to model the hydrogen sensing capabilities of pure ZnO and ZnO nanocomposites containing various additives. The GR model predicted 297 experimental responses from 10 ZnO-based sensors, achieving a mean absolute error of 0.29, a relative absolute error of 1.56%, and a regression coefficient of 0.9977. These results demonstrate superior accuracy compared to other neural network models such as cascade feedforward, radial basis, Elman recurrent, and multilayer perceptron networks.

**Fig. 16.**
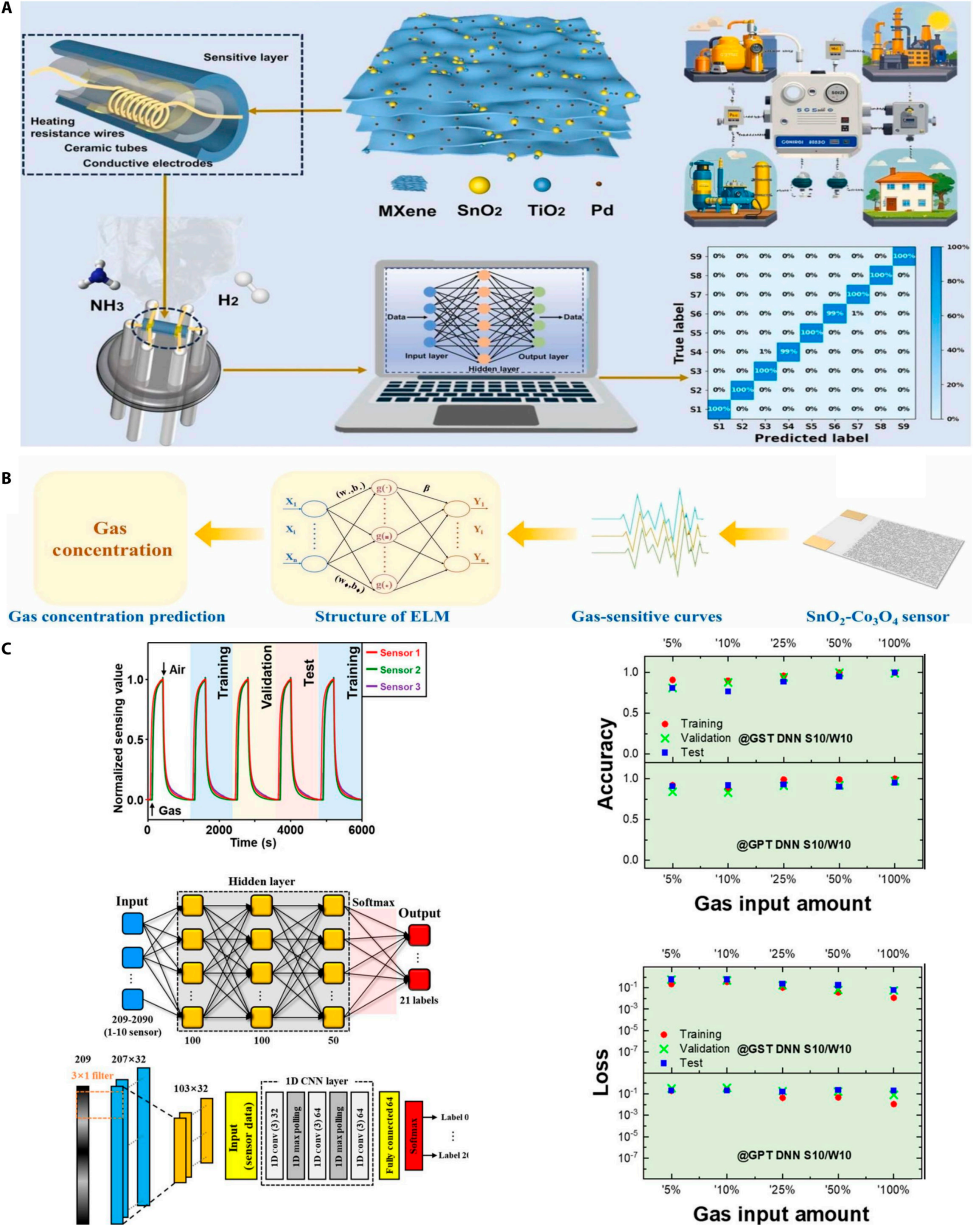
Research on ML algorithms for hydrogen sensors in recent years. (A) ML enhances the hydrogen gas sensing of SnO_2_-TiO_2_ heterojunctions on MXene [111]. (B) SnO_2_-Co_3_O_4_ nanocomposite sensor [112]. (C) Synergistic integration of ML with SnO_2_ and WO_3_ breath sensors [113].

At the same time, let us focus on the advancements in hydrogen identification, classification, or prediction brought about by recent research in ML algorithms. As shown in Fig. 16A, this study by Zhang et al. [111] presents an important advancement in hydrogen sensing by integrating ML techniques into a novel SnO₂-TiO₂/MXene-based sensor system. Through the use of algorithms such as SVM and ANN, the sensor achieves enhanced selectivity, sensitivity, and accurate quantitative prediction of hydrogen concentrations. The application of ML enables the system to reliably interpret complex sensing signals and improves overall performance beyond traditional gas sensing approaches, highlighting its potential for intelligent and precise hydrogen detection. Qin et al. [112] presents a SnO₂-Co₃O₄ nanocomposite sensor with outstanding hydrogen selectivity in mixed gas environments, as shown in Fig. 16B. Notably, it introduces ML models—particularly extreme learning machine (ELM) and multilayer perceptron (MLP)—to accurately predict hydrogen concentrations under CO interference. Among them, the ELM model achieves high prediction accuracy with minimal error and fast training time, enhancing the sensor’s reliability in complex gas mixtures. This integration of ML provides a powerful solution to the selectivity limitations of traditional MOS sensors. In addition, as shown in Fig. 16C, the research by Nam et al. [113] presents a hydrogen detection strategy that integrates materials engineering with advanced ML algorithms. This is achieved by constructing a hybrid gas-sensitive system based on an array of SnO₂ and WO₃ sensors, incorporating various supervised learning methods, including DNN and CNN, to accurately identify and differentiate hydrogen from other volatile gases such as acetone and ethanol. This study constructs a recognition framework with high nonlinear mapping capability through the heterogeneous integration of sensor materials and the synergistic training of ML models. Notably, in the face of complex mixed atmospheres and high humidity challenges, the use of DNN for extracting time-series response features, combined with CNN for feature learning from response curve images, enhances the accuracy and selectivity of hydrogen detection.

## Conclusions and Outlook

Hydrogen sensors are becoming increasingly crucial with the growing utilization of hydrogen gas as both an energy source and a chemical reactant. This study explores advancements in 4 key areas: At the nanoscale, the incorporation of nanohybrid structural innovations has led to the development of novel sensor architectures that effectively enhance hydrogen detection efficiency and accuracy. Additionally, the advancement of optical hydrogen sensors and next-generation hybrid functional mechanisms has effectively boosted both the precision and adaptability of these sensors. Optical hydrogen sensors provide high sensitivity, accurate detection, and robust resistance to electromagnetic interference. New hybrid detection models, including self-powered and long-range sensors, leverage diverse material properties, further enhancing sensor capabilities. Moreover, the emergence of modern ML techniques has provided effective solutions for processing hydrogen sensor data, thereby improving detection accuracy. This paper underscores the role of ML in advancing hydrogen sensor performance, promising faster and more precise detection in the future. From a personal perspective, the key challenge in the development of hydrogen sensors lies in the contradiction between the intrinsic properties of materials and system integration: How to effectively embed high-performance nano-hybrid materials into low-power, intelligently recognizable systems remains a technical bottleneck. The most urgent research demand is to promote hydrogen sensing technology toward intelligence, integration, and practical application through a collaborative design approach that encompasses materials, devices, systems, and algorithms. In conclusion, the integration of nanohybrid technologies, optical sensing, hybrid mechanisms, and ML represents substantial progress in improving the functionality, reliability, and flexibility of hydrogen sensors. These technological advancements offer critical solutions for ensuring hydrogen safety in high-risk or sensitive environments. The advantages and applications of various sensors are summarized in Table 2.

**Table 2.** Summary of typical features of various hydrogen sensors

Sensor type	Advantages	Disadvantages	Applications
Catalytic	High sensitivity; fast response	Sensitive to temperature; poisoning of catalyst	Combustible gas detection, environmental monitoring, industrial process control
Electrochemical	Good sensitivity and selectivity; direct electrical signal output	Requires reference and auxiliary electrodes; susceptible to electrolyte interference	Health monitoring, environmental monitoring, industrial safety
Resistance based	Simple structure; low cost; high sensitivity	Poor selectivity; sensitive to environmental factors (e.g., humidity)	Domestic and industrial gas leak detection, air quality monitoring
Optical	Immune to electromagnetic interference; durability and stability	High manufacturing costs; relatively lower sensitivity	Remote sensing, hazardous area gas detection (e.g., oil wells, chemical plants)
Surface acoustic wave	Does not require power; operates in harsh environments	Lower sensitivity and selectivity; affected by background noise	Industrial process monitoring, large facility structural health monitoring
Thermoelectric	Can measure very low concentrations of hydrogen; no external power required	Sensitivity affected by temperature; longer response and recovery times	Environmental monitoring, scientific research
Mechanical	Simple structure; good stability	Generally lower sensitivity and response speed compared to other types; affected by mechanical vibration and shock	Industrial safety detection, early warning for hydrogen leakage in mechanical equipment

The future of hydrogen gas sensing is anchored in the ongoing exploration of innovative materials, including 2D materials beyond graphene, MOFs, and conductive polymers, which promise to deliver unparalleled sensing capabilities. The integration of hydrogen sensors with IoT technology will enable real-time monitoring and intelligent data analysis, broadening their utility in safety management, environmental surveillance, and industrial process control. Nevertheless, the scalability and cost-effectiveness of sensor fabrication remain critical factors for widespread adoption. Advances in fabrication techniques such as printable electronics and microfabrication may prove essential for producing high-performance, low-cost sensors on a large scale. Moreover, the convergence of hydrogen gas sensors with ML presents a promising avenue for enhancing safety, efficiency, and predictive capabilities across various applications. ML algorithms can analyze complex datasets generated by these sensors to uncover patterns, trends, and anomalies that might not be immediately discernible. This synergy could facilitate the development of smart sensors with adaptive and predictive functionalities, thereby improving both detection accuracy and response times to hydrogen leaks. Furthermore, it paves the way for creating autonomous monitoring systems that are capable of continuous learning and adaptation to their environments. As research progresses in addressing existing challenges while exploring new possibilities, the next generation of hydrogen gas sensors is poised to offer even greater sensitivity, selectivity, and functionality—ultimately contributing to safer and more sustainable utilization of hydrogen energy.

## Data Availability

All of the data that support the findings of this study are reported in the main text and the Supplementary Materials. Source data are available from the corresponding author on reasonable request.
